# Genetic Landscape of Nonobstructive Azoospermia and New Perspectives for the Clinic

**DOI:** 10.3390/jcm9020300

**Published:** 2020-01-21

**Authors:** Miriam Cerván-Martín, José A. Castilla, Rogelio J. Palomino-Morales, F. David Carmona

**Affiliations:** 1 Departamento de Genética e Instituto de Biotecnología, Universidad de Granada, Centro de Investigación Biomédica (CIBM), Parque Tecnológico Ciencias de la Salud, Av. del Conocimiento, s/n, 18016 Granada, Spain; mcervan@ugr.es; 2 Instituto de Investigación Biosanitaria ibs.GRANADA, Av. de Madrid, 15, Pabellón de Consultas Externas 2, 2ª Planta, 18012 Granada, Spain; josea.castilla.sspa@juntadeandalucia.es (J.A.C.); rpm@ugr.es (R.J.P.-M.); 3 Unidad de Reproducción, UGC Obstetricia y Ginecología, HU Virgen de las Nieves, Av. de las Fuerzas Armadas 2, 18014 Granada, Spain; 4 CEIFER Biobanco—NextClinics, Calle Maestro Bretón 1, 18004 Granada, Spain; 5 Departamento de Bioquímica y Biología Molecular I, Universidad de Granada, Facultad de Ciencias, Av. de Fuente Nueva s/n, 18071 Granada, Spain

**Keywords:** male infertility, azoospermia, genetic component, mutations, SNPs

## Abstract

Nonobstructive azoospermia (NOA) represents the most severe expression of male infertility, involving around 1% of the male population and 10% of infertile men. This condition is characterised by the inability of the testis to produce sperm cells, and it is considered to have an important genetic component. During the last two decades, different genetic anomalies, including microdeletions of the Y chromosome, karyotype defects, and missense mutations in genes involved in the reproductive function, have been described as the primary cause of NOA in many infertile men. However, these alterations only explain around 25% of azoospermic cases, with the remaining patients showing an idiopathic origin. Recent studies clearly suggest that the so-called idiopathic NOA has a complex aetiology with a polygenic inheritance, which may alter the spermatogenic process. Although we are far from a complete understanding of the molecular mechanisms underlying NOA, the use of the new technologies for genetic analysis has enabled a considerable increase in knowledge during the last years. In this review, we will provide a comprehensive and updated overview of the genetic basis of NOA, with a special focus on the possible application of the recent insights in clinical practice.

## 1. Introduction

Infertility is becoming a growing global public health issue according to the World Health Organization (WHO, Geneva, Switzerland), which is warning about the relevant impact that this condition has on society and the economy. Despite the difficulty in estimating the prevalence of male and female contributions to infertility, it has been reported that about 50 million couples worldwide may be affected, with male infertility contributing to half of those cases approximately [1,2]. Nonobstructive azoospermia (NOA) is considered the clinically most severe expression of male infertility, affecting around 1% of the male population and 10% of infertile men [3,4,5,6,7]. This condition is defined by a complete lack of sperm cells in the ejaculate without any reproductive tract obstruction, likely related to a failure in the spermatogenic process [8]. However, such a phenotype has extremely diverse causes (influencing different processes like gonad differentiation, the hypothalamic–pituitary axis function, and spermatogenesis), making this a highly heterogeneous disease [9]. Single genetic anomalies (including chromosome aberrations and point mutations) can explain around 25% of NOA patients, with the remaining patients being classified as idiopathic [10]. In this regard, cumulating knowledge clearly suggests that idiopathic NOA has a multifactorial aetiology, in which both environmental and genetic factors may contribute to disease development. In these cases, the genetic predisposition is likely conferred by common variations of the human genome, mostly single-nucleotide polymorphisms (SNPs) and copy number variants (CNVs), which complicate the elucidation of the underlying pathological mechanisms [9]. Identifying the genetic risk factors of NOA would definitively help urologists to improve both care and counselling of affected men.

This review aims to summarise the current knowledge on the genetic background of NOA, with emphasis on the potential impact that the recent insights could have in the management of affected individuals who hope to father a biological child.

## 2. The Spermatogenic Process

The testis represents a crucial organ for male individuals, since it is responsible for androgen production and generation of sperm cells that will carry the genetic inheritance to the offspring. This organ develops from a gonadal primordium that has the potential to differentiate either as testis or ovary. As a consequence, the molecular network underlying gonad development and function is extremely complex, and subtle alterations may produce very relevant clinical phenotypes [11].

**Figure 1:**
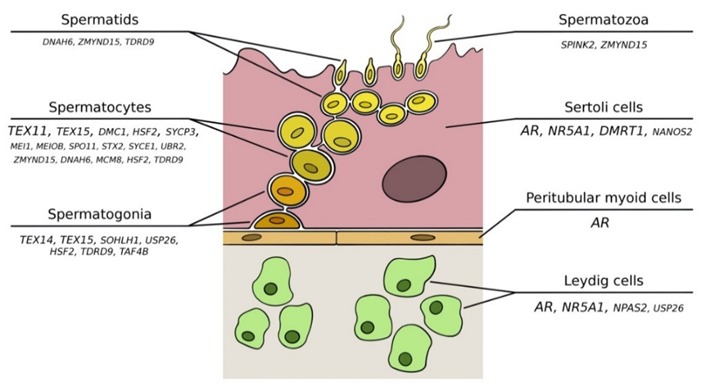
Schematic illustration of spermatogenesis. Genes with reported mutations associated with nonobstructive azoospermia are shown in the cells in which they are expressed. The font size correlates with the strength of the evidence.

After puberty, hundreds of millions of male gametes are generated daily within the wall of the seminiferous tubules in a process known as spermatogenesis [12]. This process implies a series of events in a stepwise fashion, including several division rounds of diploid spermatogonial stem cells (SSCs), meiosis of spermatocytes, and morphological differentiation of spermatids (Figure 1). Once all these steps are completed, mature spermatozoa are released into the lumen of the tubule so they can be stored in the epididymis until ejaculation. In humans, the whole process is estimated to take around three months, and it requires a delicate physical and molecular interaction between germ cells and Sertoli cells, which are the somatic supporting cells of the tubule. Other somatic cells outside the tubule, such as Leydig and peritubular-myoid cells, are also important for this cross-talk [13,14].

Different populations of spermatogonia have been described, all of them residing in the innermost part of the tubule wall. Molecular markers common to all SSCs (e.g., FOXO1, CDH1, ZBTB16, SALL4, and LIN28) as well as SSC type-specific markers (e.g., ID4, PAX7, BMI1, GFRa1, and NANOS2) have been reported [15]. Once the differentiation program has been initiated, a self-renewing SSC divides asymmetrically to maintain the undifferentiated pool and to generate a new spermatogenic lineage (A-aligned spermatogonia). At this step, a genetic commitment to complete the spermatogenic process has been initiated, leading to differentiation to A1 spermatogonia, which are characterised by the expression of the cKIT receptor. Subsequently, A1 spermatogonia undergo several mitotic divisions generating A2, A3, A4, intermediate and B spermatogonia, and primary spermatocytes that will initiate meiosis to produce haploid secondary spermatocytes. Finally, biologically functional male gametes will be produced by morphological maturation of secondary spermatocytes, which will differentiate into spermatids and spermatozoa in a process known as spermiogenesis [16].

Because of the high relevance and complexity of the spermatogenesis process, a large number of molecules are involved in its regulatory mechanisms, such as the GnRH/FSHLH, GDNF/RET/GFRA1, FGF2/MAP2K1, CXCL12/CXCR4, retinoic acid, androgen and oestrogen signalling pathways [16,17,18]. Indeed, it has been estimated that more than 2000 genes may be involved in the maintenance of the germ cell population and satisfactory accomplishment of meiosis [19]. Besides, epigenetics and DNA integrity have been also shown to play relevant roles in this process [20,21]. Therefore, alterations of such mechanisms may result in the development of infertility issues [10].

## 3. Monogenic Causes of Nonobstructive Azoospermia

Around 10% of male infertility diagnoses are due to azoospermia [22]. Known causes of azoospermia include endocrine and chronic diseases that affect the hypothalamic–pituitary–gonadal axis (e.g., hypogonadism or diabetes), but also pathologic conditions that disrupt the transport of sperm through the vas deferens (e.g., maldescended testes, varicocele, malignancy, or urogenital infections) [23]. However, in one-third of azoospermia patients there is a discernible genetic anomaly responsible for spermatogenic failure [6]. In this regard, genetic diagnostic testing for azoospermia is usually based on the detection of karyotype aberrations, Y chromosome microdeletions, and cystic fibrosis transmembrane conductance regulator (*CFTR*) mutations [24].

### 3.1. Karyotype Abnormalities

It is widely known that the presence of cytogenetic defects may interfere with the spermatogenic process. In this regard, the aneuploidy rate in NOA patients is ten times higher than that observed in men with obstructive azoospermia [25]. Indeed, sex chromosome aneuploidies represent the most common cause of NOA, especially Klinefelter syndrome (47, XXY) [26,27,28]. This syndrome affects approximately 0.15% of men, which show testicular atrophy and dysfunction as a consequence of hyalinization of the seminiferous tubules at mid-puberty, with loss of germ cells and hyperplasia of Leydig cells [29]. However, although more than 95% of Klinefelter patients have an NOA diagnosis, focal spermatogenesis has been observed in cases of mosaicism 47, XXY/46, XY in the germ line, allowing these individuals to father a child by testicular sperm extraction (TESE) techniques [30].

Other structural chromosome mutations have been also associated with NOA, including sex and autosomal chromosome rearrangements, and translocations between X and Y chromosomes or between sex chromosomes and autosomes, which may involve crucial genes for spermatogenesis [28].

### 3.2. Y Chromosome Microdeletions

The key role in spermatogenesis of a genomic region within the long arm of the Y chromosome (Yq) has been known for more than four decades [31]. Different studies reported that several terminal and interstitial microdeletions of Yq were associated with NOA, identifying three distinct intervals essential for male fertility that were called azoospermia factor (AZF) a, b, and c (located in proximal, middle, and distal Yq11 subregions, respectively) [32,33]. Subsequently, with the use of more advanced sequencing methods, different breakpoint hotspots involving five major palindromes (labelled P1 through P5) were identified [34]. However, in clinical practice, the AZF nomenclature is still used to define the different deletion patterns, i.e., AZFa, AZFb (P5/proximal P1), AZFbc (P5/distal P1 or P4/distal P1), and AZFc (b2/b4) [35].

The Yq microdeletions are a consequence of intrachromosomal non-allelic homologous recombination events (due to the highly repetitive structure of the Y chromosome) [36,37,38,39], and overall they are present in 5%–10% of azoospermic patients [40]. The deleterious effect on male fertility is caused by the loss of several key regulatory genes of spermatogenesis [35]. Some of them are described below.

#### 3.2.1. USP9Y

*USP9Y* (ubiquitin-specific protease 9, Y chromosome, MIM*400005), also known as *DFFRY* or *SPGFY2*, represents one of the major AZFa genes. It encodes a member of the peptidase C19 family that confers stability and protection to ubiquitin-conjugated proteins involved in germ cell survival, through their deubiquitination [41,42]. Its expression is restricted to spermatids, and point mutations in this gene can lead to different clinical phenotypes of male infertility related to reduced mobility and concentration of sperm cells, including spermatid maturation arrest, oligospermia, or asthenozoospermia [43,44,45,46,47]. Similarly, complete loss of the gene has been associated with NOA [48,49].

#### 3.2.2. DDX3Y

The protein encoded by *DDX3Y* (DEAD/H box 3, Y-linked, MIM*400010), another AZFa gene formerly known as *DBY*, belongs to a family of ATP-dependent helicases involved in the regulation of the cell cycle through intramolecular interactions during RNA transcription, translation, and intron splicing [50]. A testis-specific expression of some *DDX3Y* isoforms has been reported, particularly in the male germ line [51,52]. Interestingly, there is a homologue *DDX3Y* in the X chromosome (*DDX3X*) that shares around 95% of the DNA sequence and seems to have similar molecular functions. However, *DDX3X* is mostly expressed during spermatid maturation and *DDX3Y* in early meiosis [53,54]. Although there is no direct evidence yet, it is likely that depletion of *DDX3Y* results in Sertoli cell-only syndrome (SCO) [35].

#### 3.2.3. EIF1AY

One of the genes located within the AZFb region is *EIF1AY* (eukaryotic translation initiation factor 1a, Y-linked, MIM*400014) [55]. Its encoded protein plays an important role in start codon recognition by the translation initiation machinery during spermatogenesis [56]. It has been suggested that the absence of *EIF1AY* expression may contribute to NOA development [57].

#### 3.2.4. RPS4Y2

RPS4 refers to a highly conserved protein family involved in mRNA binding to the ribosome [58]. In nonhuman primates, two *RPS4* genes have been described, named *RPS4X* and *RPS4Y* (located in chromosome X and Y, respectively). Interestingly, the human *RPS4Y* has two functional Y-linked paralogs, named *RPS4Y1* (ribosomal protein s4, Y-linked, 1, MIM*470000) and *RPS4Y2* (ribosomal protein s4, Y-linked, 2, MIM*400030), which makes this a unique feature compared to other ribosomal proteins [59,60]. In contrast with its X-linked homologue, *RPS4Y2* has a testis-specific expression pattern, being proposed as a key player in the post-transcriptional regulation during germ cell development [59,61]. The fact that *RPS4Y2* maps within the AZFb region makes this gene a good candidate to explain the development of male infertility traits when this genomic region is depleted [35].

#### 3.2.5. KDM5D

Another relevant AZFb gene is *KDM5D* (lysine-specific demethylase 5d, MIM*426000), also known as *JARID1D*. It encodes a conserved protein of the family of histone demethylases, which have crucial roles in the epigenetic regulation of gene expression. KDM5 proteins catalyse the removal of methyl groups from histone H3 lysine 4 methylation marks (H3K4me) in the genome, acting as transcriptional repressors [62,63]. It has been observed that KDM5D-mediated H3K4 demethylation is required for sexually dimorphic gene expression, and defects in the regulatory mechanism of this enzyme have been associated with different tumorigenic processes [64,65]. Regarding spermatogenesis, it seems that KDM5D is directly involved in chromatin remodelling and condensation during meiosis, acting in a cooperative manner with the MSH5 DNA repair factor [66,67].

#### 3.2.6. DAZ

Because of the evolutionary history of the sex chromosomes, the Y chromosome contains several ampliconic regions (male specific-sequences of the Y chromosome enriched with large segmental duplications that involve frequently complex structural arrangements), most of them located in the AZFc region, which have been evolved from X chromosome or even autosome counterparts [68]. The amplicons are arranged as direct repeats, inverted repeats, or palindromes, giving rise to multiple copies of the so-called “ampliconic genes”, which are prone to undergo processes of intra-chromosomal recombination events, in a process known as “gene conversion”. Interestingly, these multicopy genes show variations in their sequence, structure, and copy number amongst individuals [69]. As a consequence of this large sequence identity, deletions involving the AZFc region represent the most common Yq microdeletions (more than 80%) and have been directly involved with NOA development [39,70]. One of these multicopy gene families is *DAZ* (deleted in azoospermia, MIM*400003), which evolved from the autosomal gene *DAZL* (deleted in azoospermia-like, MIM*601486) [71]. *DAZ* has four copies distributed into two different clusters (*DAZ1/2* and *DAZ3/4*), and their expression is restricted to the testis [72]. It encodes an RNA-binding protein involved in RNA translation during sexual differentiation of XY germ cells, acting as antagonist of NANOS, another essential protein required for meiosis initiation [73]. Conditional inactivation of *Dazl* in the gonads of mouse models leads to complete absence of gamete production, highlighting the essential role of these proteins in gametogenesis [74].

Other multicopy gene families (involving ampliconic regions of the Y chromosome) potentially related to spermatogenesis and, therefore, are a candidate for NOA development include *TSPY* (testis-specific protein, Y-linked, MIM*480100, with 35 copies), *VCY* (variably charged, Y chromosome, MIM*400012, with 2 copies), *XKRY* (XK-related protein on Y chromosome, MIM*400015, with 2 copies), *CDY* (chromodomain protein, Y chromosome, MIM*400016, with 4 copies), *HSFY1* (heat-shock transcription factor, Y-linked, MIM*400029, with 2 copies), *RBMY* (RNA-binding motif protein, Y chromosome, MIM*400006, with 6 copies), *PRY* (PTPBL-related gene on Y, MIM*400019, with 2 copies), and *BPY2* (basic protein, Y chromosome, 2, MIM*400013, with 3 copies) [35].

### 3.3. Autosomal Monogenic Factors

The widespread application of novel technologies for genetic investigation of human disorders, such as next-generation sequencing (NGS), has allowed the identification of a large number of mutations in putative male infertility genes [75]. However, because of the lack of validation studies in most cases (and the considerably lower incidence of known monogenic alterations in male infertility compared to chromosomal abnormalities), the routine for genetic diagnostic testing has remained unaltered during the last two decades. Current genetic tests are based on karyotyping, analysis of the AZF region, and the screening of gene mutations associated with congenital hypogonadotropic hypogonadism (CHH, a very rare condition characterised by gonadotropin deficiency and low levels of sex steroid hormones) and obstructive azoospermia, being effective only in around 20% of azoospermic men [76]. Some of the described NOA genes with a potential value as diagnostic markers for NOA are summarised in this section.

#### 3.3.1. AR

*AR* (androgen receptor, MIM*313700), also known as *DHTR* (dihydrotestosterone receptor), represents the only gene that is currently considered for genetic testing and counselling in the diagnosis of NOA [10]. *AR* is an X-linked gene that encodes a transcription factor of the steroid-hormone activated receptor family, which regulates the expression of androgen-responsive genes upon binding to the hormone ligand [77]. Androgens are essential steroid hormones for male sex development as well as the maintenance of male reproductive organs and spermatogenesis, through binding to AR [78,79].

The total number of reported *AR* mutations exceeds 1000 (many of them representing non-synonymous mutations altering the DNA binding site), with a wide spectrum of associated phenotypes ranging from the complete androgen insensitivity syndrome (phenotypic female 46, XY individuals) to a mild form of androgen insensitivity in azoospermic males with apparently normal testes [80].

Mutant mice for this gene exhibit pathological phenotypes similar to those observed in humans [81]. Interestingly, conditional knock-out mice in which *Ar* was selectively depleted in Sertoli cells showed meiotic arrest in spermatocytes, thus highlighting the relevant role that androgens may have for the spermatogenic function [82].

*AR* is a highly polymorphic gene that contains two variable number tandem repeats (VNTRs), (CAG)_n_ and (CGN)_n_ (encoding for glycine and glutamine, respectively) in the N-terminal domain (located in exon1) [83]. The length of the CAG repeats has been associated with different human disorders, including impaired sperm production and male infertility [84]. In this regard, the possible implication of the CAG repeat in the development of NOA has been extensively investigated [85]. Although the results have been controversial in some cases, it has been proposed that long CAG repeats may increase the predisposition of male infertility due to impaired androgen function [86,87].

#### 3.3.2. NR5A1

The protein encoded by *NR5A1* (nuclear receptor subfamily 5, group A, member 1, MIM*184757), also known as *SF1* and *AD4BP*, belongs to the family of nuclear receptors, which has a central role in many aspects of tissue development and function [88]. The expression of this gene was initially located in the main steroidogenic tissues of the adult mouse (adrenal cortex, testis, and ovary), related to the synthesis of steroids. Shortly after, it was discovered that it also played an important role in the synthesis of gonadotropins as well as in sex differentiation and development of both the gonad and the adrenal gland [89,90,91,92,93,94]. Indeed, the SF-1 protein is a transcription factor that modulates the timing and expression levels of many target genes, including key players of the hypothalamic–pituitary–steroidogenic axis [95].

Mutations of *Nr5a1* in mouse models cause abnormal development of the hypothalamus and pituitary gland, showing absence of both luteinizing and follicle-stimulating hormones [93]. Knock-out mice are born at the expected age, but shortly after birth they die due to adrenocortical insufficiency. These mice lack adrenal glands and gonads, and XY individuals develop Mullerian ducts resulting in internal female genitals [94,96].

In humans, different clinical conditions have been associated with autosomal dominant mutations of *NR5A1*, including primary adrenal insufficiency, primary ovarian insufficiency, and different alterations of male sexual development, such as 46, XY sex reversal, anorchidism, hypospadias, testicular dysgenesis, and spermatogenic failure [97].

Regarding NOA, in 2010 Bashamboo and colleagues [98] screened the coding sequence of *NR5A1* in a population of mixed ancestry composed of 315 men with idiopathic spermatogenic failure and 729 healthy controls (359 normozoospermic men and 370 males with self-reported paternity of at least two children). Interestingly, they identified heterozygous missense mutations in four azoospermic individuals (3.9% of all azoospermic men analysed) and two patients with severe oligospermia (4.3% of men with this phenotype). Noteworthy, the control population did not show any rare variation, and the described mutations were not found in more than 2100 additional control samples.

Three years later, the Tuttelmann’s group conducted a similar study in a German cohort of 478 patients with diagnosis of spermatogenic failure (270 with NOA and 218 with severe oligospermia) and 237 men with normal semen parameters [99]. The authors identified three additional heterozygous missense mutations with predicted pathogenicity in protein function (one of them in an NOA patient). Several synonymous mutations were also observed, although they were present in some control individuals.

In 2015, two studies reported additional heterozygous *NR5A1* non-synonymous mutations in NOA patients. Ferlin and collaborators [100] observed seven novel *NR5A1* missense mutations in Italian subjects with severe spermatogenic impairment, three of them carried by NOA patients (one with idiopathic NOA and two with unilateral cryptorchidism). Similarly, two Iranian NOA patients were reported to harbour heterozygous *NR5A1* missense mutations out of 90 azoospermic patients analysed [101]. In either study, no mutations were detected in fertile individuals, with or without history of cryptorchidism [100,101].

In contrast with the previously mentioned studies, in a recent screening on the Indian population, no mutations within *NR5A1* were detected in 414 NOA patients [102].

#### 3.3.3. DMRT1

*DMRT1* (doublesex- and MAB3-related transcription factor 1, MIM*602424) belongs to the *DMRT* family, which encodes a group of highly conserved transcription factors involved in sex determination and gonadal development of several metazoan phyla [103]. This gene family represented the first described case of sex regulatory genes in both vertebrates and invertebrates [104,105]. *DMRT1* expression is detected in pre-Sertoli cells of the undifferentiated gonads shortly after male sex determination (at the sixth week of pregnancy). Its expression remains active until adulthood, representing a Sertoli cell marker [106].

Subtelomeric deletions of the short arm of the human chromosome 9 (in which *DMRT1* is located) have been associated with 46, XY sex reversal and gonadal dysgenesis in XY individuals [107]. Interestingly, smaller deletions encompassing *DMRT1* were identified in five infertile men with azoospermia but no symptoms of gonadal dysgenesis.

Unexpectedly, the experimental removal of *Dmrt1* from the mouse genome caused no abnormalities in either sex determination or the embryonic gonadal development. However, the testes of those mice during postnatal development underwent rapid deformation due to a failure in the differentiation of Sertoli cells, which showed uncontrolled proliferation and, finally, apoptosis. The germ cells, in turn, did not migrate to the periphery of the seminiferous tubules and died also shortly after birth [108].

In relation to NOA, in 2013 Lopes and colleagues [109] identified deletions of the *DMRT1* exonic sequence in five subjects diagnosed with idiopathic NOA (two Europeans and three Han Chinese) from a total cohort of 1423 azoospermic patients (323 Europeans and 979 Chinese) and 2834 fertile controls (1100 Europeans and 1734 Chinese), through the analysis of large-scale genotyping data. Interestingly, one of these individuals showed SCO.

One year later, Tewes and colleagues [110] analysed the *DMRT1* sequence in 131 individuals diagnosed with NOA and 215 normozoospermic controls, reporting two cases of NOA (one of them with SCO) that showed heterozygosity for a putative pathogenic transition mutation in the third exon of the gene. The *DMRT1* mutation observed in the SCO patient was detected in another NOA individual from Brazil (from a total of 16 azoospermic patients analysed) in a recent study [111].

Finally, in 2015, the Lopes’ group identified three additional noncoding variants located in regulatory regions of *DMRT1*, by analysing a Portuguese study cohort of 155 NOA and 376 controls (75 normozoospermic and 301 with self-reported fatherhood) using a multiplex ligation probe assay and Sanger sequencing [112]. One of them, located in the promoter region, showed clear evidence of a key regulatory role in *Dmrt1* repression. In addition, the frequency of some coding and noncoding *DMRT1* rare variants were also significantly higher in the NOA group compared with the control one.

#### 3.3.4. TEX11

*TEX11* (testis-expressed gene 11, MIM*300311) was discovered in 2001 through cDNA subtraction of specific transcripts of mouse spermatogonia that were not present in somatic cells [113]. It encodes a highly conserved meiosis-specific protein involved in the assembly and maintenance of the synaptonemal complex during chromosome recombination in prophase I. Indeed, mutant mice for *Tex11* exhibited achiasmate chromosomes as well as impaired double-strand break repair and chromosomal crossover [114]. Notably, *TEX11* is an X-linked gene, which indicates the important role that this sex chromosome may have in spermatogenesis.

In 2015, Yatsenko and colleagues [115] reported a hemizygous deletion of three exons of *TEX11* (encoding a fragment of the meiosis-specific domain SPO22) in two NOA patients. In a subsequent step, the authors performed a comprehensive mutation screening that revealed additional *TEX11* mutations, including non-synonymous changes of the coding sequence and splicing mutations, in 7 out of 289 analysed NOA individuals. Interestingly, five of these patients harbouring *TEX11* mutations had a meiotic arrest (representing 15% of all patients with meiotic arrest in the study population) that resembled the abnormal phenotype observed in mutant mice [114]. No single mutation was observed in any SCO patient or in 384 normozoospermic controls. In addition, the authors confirmed the expression of *TEX11* in late-pachytene spermatocytes and in spermatids, being completely absent in Sertoli cells [115].

The high relevance of *TEX11* in NOA was further confirmed in different independent sequencing studies [116,117,118,119], as well as in gene expression studies that reported a downregulation of *TEX11* in testis samples from MA patients compared to controls [120].

#### 3.3.5. TEX14 and TEX15

*TEX14* (testis-expressed gene 14, MIM*605792) and *TEX15* (testis-expressed gene 15, MIM*605795) represent two additional spermatogonium-specific genes identified by Wang et al. [113]. However, contrary to *TEX11*, these two genes have an autosome location (chromosomes 17q22 and 8p12, respectively), implying a double dosage in males. *TEX14* encodes a protein kinase that is expressed almost exclusively in male spermatogonia, in which it seems to regulate their differentiation [121,122,123], whereas *TEX15* encodes a protein with a function similar to that of *TEX11* (double-strand DNA break repair and chromosomal synapsis) that has been detected in both testis and ovaries [124]. Knock-out mice for these two genes show spermatogenic failure at different levels (disruption of spermatogenesis before the completion of the first meiotic division in *Tex14* null mice, and meiotic arrest in *Tex15* null mice) [121,124].

In humans, different mutations of both genes have been recently described in NOA patients. Two NOA brothers contained a 10 bp deletion within *TEX14* that led to a truncated protein. Consistent with the observations in animal models, the germ line population of both brothers consisted mostly of undifferentiated spermatogonia [125]. In addition, Fakhro and collaborators [123] used whole-exon sequencing in eight consanguineous families and described a deleterious recessive mutation within *TEX14* that segregated with disease. Regarding *TEX15*, three studies reported mutations associated with NOA. Okutman and colleagues [126] identified a heterozygous nonsense mutation in three Turkish brothers diagnosed with NOA and showing meiotic arrest, resulting in the generation of a premature stop codon. Colombo and colleagues [127] performed exome sequencing in two infertile siblings affected by NOA, identifying another nonsense mutation and a single nucleotide deletion that led to premature stop codons in the *TEX15* locus. Finally, the recent sequencing study of 16 Brazilian patients affected by NOA by Araujo et al. [111] also reported novel rare variants in both *TEX14* and *TEX15*.

#### 3.3.6. NPAS2

*NPAS2* (neuronal PAS domain protein 2, MIM*603347) encodes a transcription factor of the basic helix-loop-helix-PAS (bHLH-PAS) family that is mostly expressed in the central nervous system, in which it seems to regulate the circadian rhythms of the forebrain by interacting with the circadian locomotor output cycles kaput (CLOCK) proteins [128,129]. In 2015, Ramasamy and colleagues [130] analysed a consanguineous Turkish family, which included three infertile brothers affected by NOA, and identified a missense mutation located in exon 14 of *NPAS2*. Notably, a family segregation was observed, as all three siblings were homozygous for the mutation, whereas the mother and a fourth, fertile brother were heterozygous. Besides, the *NPAS2* mutation was not detected in a control population of 50 fertile men. The authors proposed that the pathogenic effect of this mutation could be related to a disruption in steroidogenesis, by affecting the interaction of NPAS2 with circadian molecules like CLOCK.

#### 3.3.7. Other Putative Nonobstructive Azoospermia Genes

Additional genes involved in spermatogenesis have been also proposed as single molecular causes of NOA, but, in most cases, further functional and/or validation studies are required to confirm the original findings. Some examples include *SOHLH1* (spermatogenesis- and oogenesis-specific basic helix-loop-helix protein 1, MIM*610224) [117,131], *USP26* (ubiquitin-specific protease 26, MIM*300309) [132,133], *SYCP3* (synaptonemal complex protein 3, MIM*604759) [134], *MEIOB* (meiosis-specific protein with OD domains, MIM*617670), *DNAH6* (dynein, axonemal, heavy chain 6, MIM*603336) [125], *ZMYND15* (zinc finger MYND-containing protein 15, MIM*614312), *TAF4B* (TATA box-binding protein-associated factor, MIM*601689) [135], *SYCE1* (synaptonemal complex central element protein 1, MIM*611486) [136], *MCM8* (minichromosome maintenance complex component 8, MIM*608187) [137], *HSF2* (heat-shock transcription factor 2, MIM*140581) [138], *SPINK2* (serine protease inhibitor, KAZAL-type, 2) [139], and *TDRD9* (TUDOR domain-containing protein 9, MIM*617963) [140], amongst others [75] (Figure 1).

## 4. Common Variation Associated with Susceptibility to Nonobstructive Azoospermia

Despite the great progress made during the golden era of NGS in the elucidation of the genetic causes of azoospermia, there remains a considerably large proportion of missing heritability that still needs to be accounted for. Indeed, in a large proportion of patients with spermatogenic disturbances, the aetiology remains unknown [141]. Many studies have been performed to shed light into the so-called idiopathic NOA, but, in most cases, they have been strongly limited by low sample sizes and heterogeneous inclusion criteria of the study groups [85,142] (Table 1).

### 4.1. Candidate Gene Approach

During the last three decades, big efforts have been made to identify candidate gene polymorphisms associated with a complex form of idiopathic NOA. SNPs have been the most analysed variations, with some studies on VNTRs and CNVs. Selected genes include those involved in hormone production, regulation of the cell cycle, and spermatogenesis [85,142]. Although some significant genetic associations with spermatogenic failure have been published (mainly within regulatory genes of meiosis), most candidate gene studies have been performed in Asian populations and either lack validation in replication cohorts or show conflicting results (Table 1). The main reasons for such inconsistency are (1) the extremely reduced case/control cohort sizes analysed, which had led most likely to many type I and II errors in statistical hypothesis testing, and (2) the poor clinical characterisation of the patients included. Replicated associations of NOA-specific candidate genes include *AR* (see above) [143,144], *PIWIL4* (PIWI-like 4, MIM*610315, encoding a key molecule for retrotransposon silencing in the germ line) [145,146,147], *MTHFR* (methylenetetrahydrofolate reductase, MIM*607093; an important regulatory gene involved in folate metabolism) [148,149,150], *MTR* (5-methyltetrahydrofolate-homocysteine S-methyltransferase, MIM*156570; responsible for the regeneration of methionine from homocysteine by transferring of a methyl group) [149,151], *NOS3* (nitric oxide synthase 3, MIM*163729; involved in the release of nitric oxide for the regulation of the reproductive function) [152,153], and *H2BFWT* (H2B histone family, member W, testis-specific, MIM*300507, a testis-specific histone variant gene related to spermatogenesis) [154,155,156].

### 4.2. Insights from Large-Scale Genetic Studies

The development of high-throughput genotyping platforms was a breakthrough that allowed the investigation of the genetic basis of complex traits at a genome-wide level [195]. In particular, the genome-wide association studies (GWAS), in which genetic variation across the whole genome is interrogated in a hypothesis-free fashion, have represented a major advance in biomedical discovery [196]. However, as described below, the GWAS strategy has not been very useful to define the genetic component of NOA, mainly because of the strong limitations of the study designs.

#### 4.2.1. Pilot GWAS in Europeans

The first GWAS of NOA was conducted in 2009 by Aston and Carrell [197]. In this study, 370,000 SNPs were analysed in 80 patients with spermatogenic impairment (52 with severe oligospermia and only 40 with NOA) and 80 normozoospermic controls of European descent. Such a small study cohort implied a statistical power close to zero, which prevented the authors from obtaining any relevant results. It should be noted that hundreds of thousands of independent tests are conducted in a GWAS. Hence, a *p*-value threshold for statistical significance of 5 × 10^−8^ is required to control the (highly probable) type I errors in these types of studies [198,199,200]. Therefore, considerably large case/control populations are needed to discern disease-specific associations from the numerous false positive signals. Taking this into consideration, the authors performed a follow-up study in a slightly larger European population composed of 141 severe oligospermic individuals, 80 NOA patients, 63 moderately oligospermic individuals, and 158 normospermic controls. They analysed 172 candidate SNPs (based on both gene function and previously published reports of association with male infertility) and performed a combined analysis with their previous GWAS data [161]. The comparison of the NOA group against the control one evidenced suggestive associations just for two non-synonymous SNPs (rs34605051 and rs10246939) of the genes *KDM3A* (lysine-specific demethylase 3A, MIM* 611512; a histone demethylase involved in packaging and condensation of sperm chromatin) and *TAS2R38* (taste receptor, type 2, member 38, MIM*607751; a surface protein of taste receptor cells with no reported function in the testis). Other SNPs showed significant P-values when NOA and severe oligospermia patients were analysed together, including two missense variants of *TEX15* (rs323344 and rs323345) [161].

#### 4.2.2. First GWAS in East Asians

The first well-powered GWAS was published in 2011 by Hu et al. [162]. The authors performed a three-stage study comprising a total of 2927 NOA patients and 5734 control subjects, all from the Han Chinese population. This study reported the first NOA associations at the genome-wide level of significance in the genomic regions of *PRMT6* (protein arginine *N*-methyltransferase 6, MIM*608274; a methyltransferase expressed in spermatogonia), *PEX10* (peroxisome biogenesis factor 10, MIM*602859; a peroxisomal protein potentially involved in spermatogenesis), and *SOX5* (SRY-BOX 5, MIM*604975; encoding a transcription factor restricted to post-meiotic germ cells during spermatogenesis). *SOX5* was also associated to oligospermia in a subsequent study [201]. However, the NOA population of the GWAS included individuals with partial deletions of the AZF region, and the controls were not healthy (they were obtained from a lung cancer study) and lacked semen analysis, which could have influenced the final results [162]. Indeed, inconsistent results were obtained in different replication studies. Whereas the three NOA associations described in the GWAS failed to replicate in a population of 490 NOA patients and 1167 controls from Japan [202], both the *PEX10* and *SOX5* risk variants showed evidence of association with NOA in an independent study cohort from central China composed of 301 azoospermic cases and 720 normal controls [203].

In 2014, aimed to identify additional NOA susceptibility factors, the same group conducted an extended three-stage validation study of the GWAS in 3608 NOA cases (including the original GWAS cohort) and 5909 controls from China [165]. Genetic associations at the genome-wide level of significance were observed in three new loci: a genomic region near *IL17A* (interleukin 17A, MIM*603149; encoding a proinflammatory cytokine), *ABLIM1* (actin-binding LIM protein family, member 1, MIM*602330; a regulatory gene of the actin-dependent signalling), and *HLA-DRA* (major histocompatibility complex, class II, DR alpha, MIM*142860; encoding the alpha subunit of the HLA-DR molecule). The two latter associations were further replicated in independent Chinese populations [204,205].

Another follow-up study of the GWAS was performed by Qin and colleagues in 2014 [166]. The authors obtained imputed genotypes from the original GWAS data and carried out a two-stage association study of 24,238 makers (selected for their location within genes of canonical pathways known to be important in spermatogenesis) in a Han Chinese case/control cohort comprising 1653 NOA cases and 2329 controls. The SNPs rs1406714 in *CHD2* (chromodomain helicase DNA-binding protein 2, MIM*602119; which may play a role in the DNA damage response and genome stability maintenance), rs2126986 in *GNAO1* (guanine nucleotide-binding protein, alpha-activating activity polypeptide O, MIM*139311; a member of guanine nucleotide binding proteins), and rs7226979 in *BCL2* (B-cell CLL/lymphoma 2, MIM* 151430; encoding an apoptosis regulator) were identified as novel NOA risk variants. However, these associations have not been replicated in independent studies so far.

#### 4.2.3. Second GWAS in East Asians

A second GWAS of NOA in the Han Chinese population was conducted by Zhao and collaborators in 2012 [163]. The study followed a three-stage approach, including a total of 2226 NOA cases and 4576 controls. Although the large sample set allowed the authors to perform the analyses with a considerably high statistical power, almost half of the control cohort included in the first stage was composed of women, representing an important limitation. Despite this, different association signals within the HLA region were associated with NOA risk at the genome-wide significance level. The strongest one corresponded to a haplotype block of the *HLA-DRA* locus, whose lead variant was rs3129878 (located in intron 1 of the gene). A second independent SNP (rs498422) located in the nearby gene *TSBP1* (testis-expressed basic protein 1, MIM*618151; encoding a protein expressed in the testis with unknown function) was also detected. The associated HLA variants were described as tag SNPs of the classical allele HLA-DRB*0405. Therefore, this study highlighted the HLA system as a relevant player in the development of idiopathic NOA, likely by triggering autoimmune/autoinflammatory responses against germ cell antigens in pathogenic situations in which the blood–testis barrier could be damaged [206].

#### 4.2.4. Fine-Mapping of the Major Histocompatibility Complex Region

**Figure 2:**
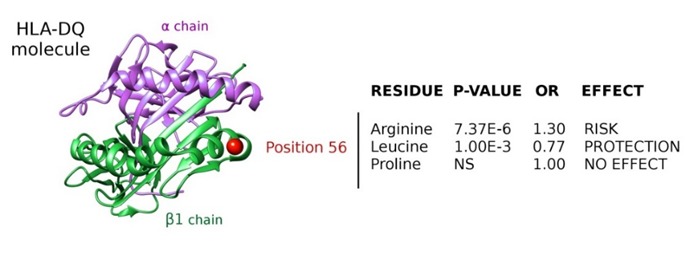
Ribbon representation of the HLA-DQ molecule. The most associated amino acid position with nonobstructive azoospermia susceptibility is highlighted with a red sphere. *P*-values, odds ratios (ORs), and the effect conferred by the different residues at that position are also shown. Data obtained from Huang et al. [193].

In a recent study, Huang and colleagues aimed to fine-map the HLA association with NOA described in the two GWASs of the Han Chinese population [193]. With that purpose, they used a specific imputation method to infer classical HLA alleles, SNPs, and polymorphic amino acid positions using the data of the extended HLA region from the GWAS by Hu et al. [162]. The most statistically significant classical haplotype was HLA Class II HLA-DRB1*13:02, which had been reported before as an NOA risk allele in the Japanese population [207]. Additionally, two SNPs explained most of the HLA association with NOA, i.e., the *HLA-DRA* polymorphism rs7194 (reported as putative association marker in the two original GWASs) and the HLA-B polymorphism rs4997052, which represented a novel risk variant for NOA. The authors estimated that the proportion of phenotypic variance of NOA explained by these two HLA SNPs and the one established in the GWAS by Zhao [163] was between 0.5% and 1%. Regarding the analysis at the amino acid level, position 55 of the HLA-DQB1 molecule represented the most statistically significant multiallelic site (Figure 2). Three possible residues were detected at that position: one of them representing a risk residue (arginine), another one representing a protective residue (leucine), and the third one with no effect on the susceptibility to NOA (proline). Other independently associated amino acid positions were HLA-DRB1*73 (with glycine conferring the higher risk) and HLA-B*166 (serine being the strongest risk residue).

#### 4.2.5. GWAS in Hutterites

Also in 2012, a GWAS of male infertility was performed in Hutterites (a North American ethnoreligious group of European ancestry that has large family sizes because contraception is proscribed) by Kosova and colleagues [208]. The authors tested for association with two heritable measures of fertility, family size and birth rate, in a random population of 269 married men. To validate the GWAS results, the most highly associated SNPs were genotyped in an independent population of 123 ethnically diverse male individuals from Chicago. However, because of the reduced statistical power of the study, no associated SNPs at the genome-wide level of significance were identified.

Subsequent candidate gene studies in Japanese were designed to evaluate the possible association with NOA susceptibility of the suggestive signals observed in the GWAS of the Hutterite population (those with P-values lower than 1 × 10^−4^). The first of them was performed in 2015 and included a total of 917 Japanese subjects (76 NOA patients, 50 patients with oligospermia, and 791 fertile men) [171]. Interestingly, an intronic variant of *EPSTI1* (epithelial stromal interaction 1, MIM*607441; encoding the epithelial stromal interaction protein 1, which is highly expressed in the testis) was associated with NOA at the study-wide level of significance after correction for multiple testing. The second study was also conducted by the same team three years later using the same case/control cohort [185]. In this case, a significant association was observed between an intronic variant of *DPF3* (D4, zinc, and double PHD fingers family, member 3, MIM*601672; a transcription regulator involved in chromatin remodelling) and NOA risk.

#### 4.2.6. Other Genome-Wide Approaches

Other large-scale approaches have been followed during the last years to shed light into the molecular causes of idiopathic NOA, including (1) an exome-wide association study that described three rare variants associated with NOA in the chromosome 6 genes *HIST1H1E* (histone gene cluster 1, H1 histone family, member E; MIM*142220; encoding the linker histone H1.4), *FKBPL* (FK506-binding protein-like, MIM*617076; which is involved in the steroid hormone signalling), and *MSH5* (MutS homolog 5, MIM*603382; which plays a role in meiotic segregation fidelity and recombination) [209]; (2) different comparative genomic hybridization (CGH) studies that identified NOA-specific recurrent CNVs such as CNV67 (on Xq28), with potential clinical application [109,210,211,212,213]; (3) miRNA profiling in the seminal plasma, in which miR-141, miR-429, and miR-7-1-3p were significantly increased in NOA patients compared with fertile controls [214]; (4) a transcriptome-wide association study that established *PILRA* (paired immunoglobulin-like type 2 receptor, alpha, MIM*605341; potentially involved in the regulation of spermatogenesis and the blood–testis barrier) and *ZNF676* (zinc finger protein 676; which may regulate the expression of genes associated with germ cell development) as putative novel NOA genes [215]; and (5) two genome-wide DNA methylation studies that identified NOA-specific methylation profiles in testicular tissues, including hyper-methylation of *SOX30* (SRY-box 30, MIM*606698; encoding an essential transcription factor for spermiogenesis) at the gene promoter, thus providing insight into the mechanisms underlying idiopathic NOA [216,217].

## 5. Clinical Relevance of the Genetic Studies of Nonobstructive Azoospermia

The rapid improvement of the new technologies for genetic analysis has enabled a substantial increase in our understanding of the aetiology of male infertility. However, the impact of such knowledge in the current genetic workup of azoospermic men is still limited, with very few novel findings incorporated to the genetic tests routinely used in clinical practice during the last two decades [24]. A genetic cause is normally identified in only 4% of infertile men, but this percentage increases to about 20% in azoospermic patients, thus highlighting the important contribution of genetic factors in the development of the most severe forms of male infertility [76]. This genetic influence is likely more relevant in NOA patients whose infertility causes remain idiopathic despite extensive laboratory evaluation.

As summarised above, increasing evidence points to idiopathic NOA as a complex and highly polygenic condition [218]. Because of that, the elucidation of its genetic component is highly demanded by thousands of urologists who have to deal with this condition on a day-to-day basis, as it may facilitate an appropriate clinical decision for enhancing fertility. Nevertheless, about 2000 genes have been proposed to be directly involved in the spermatogenic process, with more than 600 of them showing a specific expression in male germ cells [19,219,220,221]. This fact makes it extremely difficult to visualize the overall molecular picture of azoospermia, leading some authors to be skeptical about the feasibility of translating molecular genetic insights into clinical practice, especially those related to common variations of the human genome [10,222,223].

Hopefully, a more optimistic scenario will be envisioned soon. There are some promising preliminary results supporting the important role that genetic testing may have in improving both care and counselling of patients diagnosed with idiopathic NOA [9]. For instance, Tüttelmann and colleagues have recently started to regularly analyse the gene sequence of *NR5A1*, *DMRT1*, and *TEX11* in patients with idiopathic NOA, identifying causal pathogenic mutations in 5% of them and increasing the diagnostic efficiency to 25% in their study group [76]. In this sense, identifying a higher proportion of the heritability of spermatogenic impairment would definitively allow the development of broader diagnostic panels of genes, which could help (1) to achieve an accurate diagnosis, (2) to improve the selection criteria of spermatozoa for successful assisted reproduction techniques, reducing the possible genetic burden transmitted to the offspring, and (3) to estimate reliable personalized genetic scores with a prognostic value before counselling a patients to undergo testicular sperm retrieval or endocrine therapy [23,224].

## 6. Conclusions

The development and widespread use of novel tools for genetic analysis has represented a turning point in the investigation of the genetic component of male infertility. In relation to NOA, the impact has been remarkable because azoospermic individuals are at the highest risk of harbouring genetic abnormalities. Consequently, many point mutations explaining specific cases of azoospermia are being increasingly reported, including paternal and de novo mutations in key genes involved in the testicular function. However, recent estimations suggest that around 75% of cases with severe spermatogenic impairment have an idiopathic origin [225]. In this regard, as discussed in this review, different lines of evidence suggest that, apart from those point mutations (which represent undoubtedly a primary cause of NOA in many patients), common variations of the human genome, mostly SNPs and CNVs, may also have a role in the development of pathogenic mechanisms leading to spermatogenic failure. The concerns of some authors about this assumption rely on the inconsistency of some results obtained from case/control association studies. Nevertheless, it should be noted that NOA is a very heterogeneous condition, comprising different clinical entities (such as SCO, meiotic arrest, and hypospermatogenesis) with specific histological patterns and probably distinct aetiologies. This makes essential to establish stringent selection criteria for the study cohorts, which has not been possible in most studies. Besides, a low statistical power has represented an important limitation in the case/control analyses, especially in GWAS, which require considerably large sample sets to avoid type II errors. Therefore, it is imperative to reach a broad consensus on which clinical entities of NOA can be analysed in more homogeneous study groups. Certainly, this would require the establishment of large collaborative consortia that can join forces gathering well-powered case/control cohorts. Considering the current advances in assisted reproductive techniques, with most natural barriers for in vitro fertilisation and intracytoplasmic sperm injection being overcome, we need to focus now on identifying the pieces of the genetic puzzle of male infertility, which may include not only genes involved in testicular spermatogenesis, but also countless additional genes with roles in sperm maturation after exit from testes during epididymal passage. The development of effective genetic diagnostic and prognostic markers would be essential for counselling NOA patients with concerns about the causes of their infertility, the probability of success of testis sperm retrieval and in vitro fertilization, as well as the potential reproductive health of their children.

## Figures and Tables

**Table 1:** Common genetic variations associated with nonobstructive azoospermia through association studies.

Year	Risk Loci	Variant ID	Variant Type	Position (GRCh38)	Functional Annotation	Population	Cohort Size (Case/Control)	*p*-Value	OR (CI 95%)	Associated Trait	Strategy	Reference	Replication
1999	*AR*	(CAG)n	CNV	X: 67545318	Exonic	Japanese	41/48	0.0013	NA	NOA	Candidate gene	Yoshida et al. [157]	YES
2003	*MTHFR*	rs1801133	SNV	1:11796321	Missense	Italian	21/105	NA	NA	NOA	Candidate gene	Stuppia et al. [148]	YES
2006	*MEI1*	rs2050033	SNV	22:41763225	Synonymous	European/Israeli	26/121	0.027 *^,^*^,^*^,^*	NA	MA	Candidate gene	Sato et al. [158]	NO
2006	*MTR*	rs1805087	SNV	1:236885200	Missense	South Korean	174/325	0.0063 *^,^*	4.63 (1.40–15.31)	NOA	Candidate gene	Lee et al. [151]	YES
2010	*PACRG*	rs9347683	SNV	6:162728023	5′-UTR	Australian	206/156	0.009	1.60 (1.13–2.36)	NOA	Candidate gene	Wilson et al. [159]	NO
2010	*BCL2*	rs1800477	SNV	18:63318540	Missense	Han Chinese	198/183	0.01 *	0.45 (0.23–0.89)	NOA	Candidate gene	Ma et al. [160]	NO
2010	*KDM3A*	rs34605051	SNV	2:86466703	Missense	European	80/158	3.23 × 10^−3^ *^,^*	NA	NOA	GWAS follow-up	Aston et al. [161]	NO
2010	*TAS2R38*	rs10246939	SNV	7:141972804	Missense	European	80/158	7.24 × 10^−4^ *	NA	NOA	GWAS follow-up	Aston et al. [161]	NO
2011	*MTHFR*	rs1801131	SNV	1:11794419	Missense	Brazilian	55/173	0.01 *	0.34 (0.16–0.74)	NOA	Candidate gene	Gava et al. [149]	YES
2011	*PEX10*	rs2477686	SNV	1:2461209	Intergenic	Han Chinese	2927/5734	5.65 × 10^−12^	1.39 (1.26–1.52)	NOA	GWAS	Hu et al. [162]	NO
2011	*PRMT6*	rs12097821	SNV	1:106793679	Intergenic	Han Chinese	2927/5734	5.67 × 10^−10^	1.25 (1.17–1.34)	NOA	GWAS	Hu et al. [162]	YES
2011	*SOX5*	rs10842262	SNV	12:24031610	Intronic	Han Chinese	2927/5734	2.32 × 10^−9^	1.23 (1.15–1.32)	NOA	GWAS	Hu et al. [162]	YES
2012	*H2BFWT*	rs7885967	SNV	X:104013669	5′-UTR	Chinese	204/209	0.001	1.89 (1.28–2.79)	NOA	Candidate gene	Ying et al. [154]	YES
2012	*HLA-DRA*	rs3129878	SNV	6:32440958	Intronic	Han Chinese	2226/4576	3.70 × 10^−16^	1.37 (NA)	NOA	GWAS	Zhao et al. [163]	YES
2012	*TSBP1*	rs498422	SNV	6:32318984	Intronic	Han Chinese	2226/4576	2.43 × 10^−12^	1.42 (NA)	NOA	GWAS	Zhao et al. [163]	YES
2013	*ATM*	rs189037	SNV	11:108223106	5′-UTR	Chinese	229/236	0.003	1.41 (1.11–1.78)	NOA	Candidate gene	Li et al. [164]	NO
2013	*NOS3*	rs2070744	SNV	7:150992991	Intronic	Chinese	151/246	<0.001	2.52 (1.56–4.06)	NOA	Candidate gene	Ying et al. [152]	YES
2013	*NOS3*	rs61722009	INDEL	7:150997170	Intronic	Chinese	151/246	0.001	2.27 (1.39–3.72)	NOA	Candidate gene	Ying et al. [152]	YES
2014	*ABLIM1*	rs7099208	SNV	10:114894815	Intronic	Han Chinese	3608/5909	6.41 × 10^−14^	1.41(1.29–1.54)	NOA	GWAS replication	Hu et al. [165]	YES
2014	*BCL2*	rs7226979	SNV	18:63257737	Intronic	Han Chinese	1653/2329	4.50 × 10^−5^	1.21 (1.11–1.33)	NOA	GWAS replication	Qin et al. [166]	NO
2014	*CHD2*	rs140671	SNV	15:26976951	Intronic	Han Chinese	1653/2329	1.70 × 10^−4^	0.78 (0.68–0.89)	NOA	GWAS replication	Qin et al. [166]	NO
2014	*GNAO1*	rs2126986	SNV	16:56317795	Intronic	Han Chinese	1653/2329	2.30 × 10^−6^	1.28 (1.15–1.41)	NOA	GWAS replication	Qin et al. [166]	NO
2014	*HLA-DRA*	rs7194	SNV	6:32444703	3′-UTR	Han Chinese	3608/5909	3.76 × 10^−19^	1.30 (1.23–1.38)	NOA	GWAS replication	Hu et al. [165]	YES
2014	*IL17A*	rs13206743	SNV	6:52152310	Intergenic	Han Chinese	3608/5909	3.69 × 10^−8^	1.35 (1.22–1.51)	NOA	GWAS replication	Hu et al. [165]	NO
2014	*PIWIL4*	rs508485	SNV	11:94621313	3′-UTR	Spanish	22/56	0.021	NA	MA	Candidate gene	Muñoz et al. [145]	YES
2014	*SFRS4*	rs12046213	SNV	1:29186811	Intergenic	Chinese	962/1931	0.021	0.88 (0.78–0.98)	NOA	Candidate gene	Ni et al. [167]	NO
2014	*SFRS6*	rs6103330	SNV	20:43458814	Intronic	Chinese	962/1931	2.78 × 10^−3^	1.28 (1.09–1.50)	NOA	Candidate gene	Ni et al. [167]	NO
2014	*SFRS9*	rs17431717	SNV	12:120474407	Intronic	Chinese	962/1931	0.035	0.73 (0.54–0.98)	NOA	Candidate gene	Ni et al. [167]	NO
2014	*SFRS9*	rs10849753	SNV	12:120473010	Intronic	Chinese	962/1931	4.32 × 10^−3^	1.17 (1.05–1.31)	NOA	Candidate gene	Ni et al. [167]	NO
2014	*SPO11*	rs28368082	SNV	20:57335452	Missense	Iranian	58/50	0.006	6.68 (NA)	NOA	Candidate gene	Ghalkhani et al. [168]	NO
2015	*GSTP1*	rs1695	SNV	11:67585218	Missense	Chinese	361/234	0.023 *	1.48 (1.06–2.07)	NOA	Candidate gene	Xiong et al. [169]	NO
2015	*USF1*	rs2516838	SNV	1:161044580	Intronic	Chinese	361/368	0.02	1.43 (1.06–1.95)	NOA	Candidate gene	Zhang et al. [170]	NO
2015	*EPSTI1*	rs12870438	SNV	13:42906069	Intronic	Japanese	76/791	0.0059	1.92 (1.21–3.05)	NOA	Candidate gene	Sato et al. [171]	NO
2015	*FSHR*	rs6165	SNV	2:48963902	Missense	Iranian	126/86	0.001	2.06 (1.36–3.12)	NOA	Candidate gene	Gharesi-Fard et al. [172]	NO
2015	*MLH3*	rs175080	SNV	14:75047125	Missense	Chinese	244/614	<0.001	1.75 (1.27–2.41)	NOA	Candidate gene	Zhang et al. [173]	NO
2015	*MTHFR*	rs55763075	SNV	1:11790377	3′-UTR	Chinese	253/458	0.043	1.27 (1.01–1.58)	NOA	Candidate gene	Zhang et al. [174]	NO
2015	*SOHLH2*	rs1328626	SNV	13:36204635	Intronic	Chinese	361/368	0.038	0.80 (0.65–0.99)	NOA	Candidate gene	Song et al. [175]	NO
2015	*SOHLH2*	rs6563386	SNV	13:36202894	Intronic	Chinese	361/368	0.029	1.40 (1.03–1.90)	NOA	Candidate gene	Song et al. [175]	NO
2016	*HSA-miR-196a*	rs11614913	SNV	12:53991815	Intergenic	Chinese	140/486	0.009 *	1.76 (1.15–2.70)	NOA	Candidate gene	Lu et al. [176]	NO
2016	*NR3C1*	rs852977	SNV	5:143307929	Intronic	Japanese	335/410	5.70 × 10^−15^	3.20 (2.40–4.26)	NOA	Candidate gene	Chihara et al. [177]	NO
2016	*TDRD1*	rs77559927	SNV	10:114179297	5′-UTR	Chinese	342/493	0.03 *	0.73 (0.56–0.97)	NOA	Candidate gene	Zhu et al. [178]	NO
2016	*YBX2*	rs222859	SNV	17:7294475	Missense	Iranian	60/96	<0.05 *^,^*^,^*	0.23 (0.12–0.6)	NOA	Candidate gene	Najafipour et al. [179]	NO
2017	*CYP1A1*	rs4646903	SNV	15:74719300	Intergenic	South Indian	120/80	0.0001 *	3.71 (2.05–6.74)	NOA	Candidate gene	Ramgir et al. [180]	NO
2017	*FASL*	rs763110	SNV	1:172658358	Intergenic	Iranian	102/110	0.02 *^,^*^,^*	NA	NOA	Candidate gene	Asgari et al. [181]	NO
2017	*PRKDC*	rs7003908	SNV	8:47858141	Intronic	Iranian	102/214	0.03	1.51 (1.04–2.18)	NOA	Candidate gene	Jahantigh et al. [182]	NO
2017	*TNFR1*	rs767455	SNV	12:6341779	Synonymous	Iranian	108/119	<0.001	2.30 (1.58–3.36)	NOA	Candidate gene	Ashrafzadeh et al. [183]	NO
2017	*XRCC5*	rs6147172	VNTR	2:216109147	Promoter	Iranian	102/214	0.001	0.43 (0.26–0.73)	NOA	Candidate gene	Jahantigh et al. [182]	NO
2017	*XRCC6*	rs2267437	SNV	22:41620695	Intronic	Iranian	102/214	0.0002	1.94 (1.37–2.75)	NOA	Candidate gene	Jahantigh et al. [182]	NO
2018	*DICER1*	rs1057035	SNV	14:95087805	3′-UTR	Iranian	135/120	<0.05	1.49 (1.27–1.88)	NOA	Candidate gene	Moghbelinejad et al. [184]	NO
2018	*DPF3*	rs10129954	SNV	14:72683993	Intronic	Japanese	83/713	7.40 × 10^−3^	2.05 (1.21–3.46)	NOA	Candidate gene	Sato et al. [185]	NO
2018	*H2BFWT*	rs553509	SNV	X:104013293	Missense	Iranian	120/250	0.019	1.69 (1.09–2.62)	NOA	Candidate gene	Teimouri et al. [186]	NO
2018	*IL1A*	rs2071376	SNV	2:112777818	Intronic	Iranian	230/230	0.034	1.67 (1.04–2.68)	NOA	Candidate gene	Zamani-Badi et al. [187]	NO
2018	*IL1A*	rs17561	SNV	2:112779646	Missense	Iranian	230/230	<0.0001	2.59 (1.67–4.04)	NOA	Candidate gene	Zamani-Badi et al. [188]	NO
2018	*RNF212*	rs4045481	SNV	4:1096837	Synonymous	Chinese	220/248	0.003	1.50 (1.15–1.95)	NOA	Candidate gene	Yu et al. [189]	NO
2019	*ERCC2*	rs13181	SNV	19:45351661	Missense	Indo-European	541/416	0.03 *^,^*^,^*^,^*	1.59 (1.04–2.42)	NOA	Candidate gene	Singh et al. [190]	NO
2019	*FSHB*	rs10835638	SNV	11:30230805	Intergenic	German	659	0.017 *^,^*^,^*^,^*^,^*	0.20 (0.06–0.70)	TESE–	Candidate gene	Busch et al. [191]	NO
2019	*MSH3*	rs26279	SNV	5:80873118	Missense	Northwest Chinese	131/201	<0.05 *^,^*	2.62 (1.05–6.57)	NOA	Candidate gene	Zhao et al. [192]	NO
2019	*HLA-B*	rs4997052	SNV	6:31356367	Missense	Han Chinese	981/1657	2.26 × 10^−5^	1.30 (1.15–1.46)	NOA	GWAS imputation	Huang et al. [193]	NO
2020	*STAG3*	rs1727130	SNV	7:100213841	Intronic	Korean	77/245	0.039 *	1.64 (1.03–2.61)	NOA	Candidate gene	Nam et al. [194]	NO
2020	*STAG3*	rs1052482	SNV	7:100214213	3′-UTR	Korean	77/245	0.039 *	1.64 (1.03–2.61)	NOA	Candidate gene	Nam et al. [194]	NO

*p*-values of the allelic test are shown except for * dominant model of the minor allele; *^,^* recessive model of the minor allele; *^,^*^,^* genotypic (2-df) model; *^,^*^,^*^,^* codominant model; and *^,^*^,^*^,^*^,^* TTvsGG. CI, confidence interval; CNV, copy number variant; GWAS, genome-wide association study; INDEL, insertion/deletion variant; MA, meiotic arrest; OR, odds ratio; NOA, nonobstructive azoospermia; SNV, single-nucleotide variant; TESE–, unsuccessful testicular sperm extraction; UTR, untranslated region; VNTR, variable number tandem repeat.
